# The First Line Trench in Dentistry

**Published:** 1919-07

**Authors:** M. Evangeline Jordon

**Affiliations:** Los Angeles, Cal.


### THE FIRST LINE TRENCH IN DENTISTRY

BY M. EVANGELINE JORDON, D.D.S., LOS ANGELES, CAL.

For two reasons I am glad to bring my simple message to you. First, because this is a time when scientific prevention is being studied in every detail; and second, because this is children's year. President Wilson writing to the Secretary of Labor says, "Next to the duty of doing everything possible for soldiers at the front, there could be, it seems to me, no more patriotic duty than that of protecting the children, who constitute one-third of the population, and I heartily approve the plan for making the second year of the war one of united activity on behalf of children, and in that sense a children's year. I trust that the work may so successfully develop as to set up certain irreducible minimum standards for health, education and work of the American child."

The draft examinations of our first year of war have focused the attention of the public upcn the conditions of the teeth of our young men. It is too soon to get many statistics, but I'll mention a few.

Quoting from a paper by Dr. Adelaide Brown of San Francisco, read at the State Federation of Women's Clubs in Oakland, "In one group of 8,00 men, 480 were rejected because they did not come up to the standard of two pair of opposing grinders, biscuspids included with molars."

Dr. Costello of the Medical Reserve Corps of the U. S. Navy says, "27% of the rejections at the recruiting stations are for teeth which have been neglected until their condition is beyond repair. The minimum requirements of twenty sound teeth of which there must be four opposing incisors with crown and bridgework counting as sound and fair, and a man's mouth could hardly be considered in good condition without conforming to this requirement."

In Italy 50,000 soldiers have been incapacitated because of defective teeth.

We have no reliable statistics of the Germans, but we know that forty years ago in preparation for "the day" she began to do dentistry in the schools.

You remember that quite a number of years ago, Dr. Moore of Santa Barbara told us that finding the clinic results unsatisfactory, the German children were being given prophylaxis in the kindergarten. All this has brought us to realize the urgent necessity of prevention and such a prevention that we shall not bear the stigma of having a large per cent. of our picked population fail in the military examinations because of having defective teeth at an age when they should present almost perfect mouths.

In justice to the dental profession I must say that for years this has been recognized, but the politicians who have controlled our state revenues have been too short sighted to make adequate provision for preventive measures, and it still remains the work of the dental profession to build up a public opinion which will force such protection.

Caries of the teeth is distinctly a disease of childhood and must be combatted, just as ordinary smallpox was, and its extermination, while not as easy, is just as possible.

It is simply a question of education, with every practicing dentist as an instructor to his entire clientele.

The causes of early caries are several, among which prenatal conditions may play a part, serious illness or shocks to the system of the mother during the nine months of pregnancy may be so registered upon the system of the child that both physically and mentally it may be subnormal.

After birth the child who is nursed has twice as good a chance for better dentures as well as better shaped jaws. The largest percentage of children who need dental care have been bottle fed.

One of the most frequent mistakes of the past was made at the time of changing from liquid to solid food because the parent gave semi-solids which adhere to the teeth, instead of hard foods which leave the teeth polished and clean. The average American child was, and is given too large a per cent. of carbohydrates in its diet.

To quote Luther Burbank in his ''Training of the Human Plant''—''Growing children need a greater proportion of body-building foods, such as lean meats, fish, milk, some vegetables and fruits. They are often fed too great a proportion of sweet and starchy foods. A certain proportion of these are absolutely necessary, but we all know the 'starch babies' by their pale, flat, flabby, characterless faces, lustreless eyes and general lack of vitality.'' If he had been a dentist he would have added, ''and mouths with coated tongues and ropy saliva.'' He goes on to say, ''Less starchy foods and more fresh meats, with eggs, milk and some vegetables and fruits will give more vitality, a better growth, greater intelligence, better health and a better constitution, notwithstanding the belief of some of my vegetarian friends to the contrary."

Another mistake often made is to let the child begin breakfast with grape fruit or other acids followed by a starchy cereal such as cream of wheat, smothered in cream and sugar. Such children always improve in health when put upon a sensible diet.

Mastication plays an important part and children who have mushes and liquids with their meals seldom learn to masticate—a habit that must be acquired in childhood. Bolting of food has so many serious consequences, that in a short paper I will only mention one—pyorrhea. I have yet to see any one, who does not exercise his teeth and gums, who has passed middle life, without having swollen and bleeding gums.

Adenoids and enlarged tonsils also play their part in caries of the teeth.

The last to be mentioned of the causes of caries, is the neglect of the care of the teeth at home. It is surprising that in a day when so much is said of this, there should still be children without a tooth brush, but many young children are brought to me who have never had one.

When caries appear in the mouth of a child two or three years of age, unless careful repair work is done at once and the cause is discovered and removed, the first permanent molar, when it appears at six or seven years of age, has not a chance to survive.

Those of you who study radiographs know how futile it is to expect a perfect occlusion with these cornerstones of the arch missing, and I for one, speak feelingly of the suffering due to malocclusion from losing my first molars in my twelfth year.

One can not say too much of the first molar, the most valuable tooth in the mouth. "The First Line Trench,"

to use a war term. Its position makes its loss one never to be repaired.

Any one who studies the science of occlusion will readily tell you that the first molars are the most valuable teeth because they are in position for the six years when the deciduous teeth are exfoliating and the permanent teeth are taking their places in the growing arches.

Because it is often mistaken for a deciduous tooth it is neglected, for alas, there are still dentists who say, "Do not bother with them, they'll soon be lost," without stopping to explain that a carious deciduous molar does not need the six years it will approximate the permanent molar to destroy it. One year will do that sort of work. For destruction is always more rapid than prevention.

You ask the remedy and I answer—education.

Already it has been started in the schools, first by the clinic which brought relief to the suffering children and then increased health and vitality. This last year Dr. Proctor has been doing some work by lectures and tooth brush drill in our city schools, but it is a task of such magnitude that each one of the profession must lend a hand and make it his business to tell one or more people each day the need of care of the adolescent mouth while he is repairing the losses of an adult mouth.

The most beneficial treatment is to begin monthly prophylaxis not later than the fifth year. This treatment can not be too carefully emphasized.

The preponderance of mushy carbohydrates in the diet is one of the causes of so many children entering the kindergarten with their teeth broken down to the gums and small undersized jaws. One of our responsibilities is spreading the knowledge that no demineralized white bread or crackers should ever be given to a child under five. We not only need all the lime salts contained in the dark brands to build the teeth and bones, but we need exercise to develop the jaws. Children who eat tough bran bread made into toast instead of mush and who do not drink while eating, usually get the proper development of the jaws.

Mouth hygiene, to be effective, must be begun as the child enters school and not left until the first permanent molar is breaking down.

Unless you have examined the mouths of the children in a kindergarten you have no idea how many of them have from one to six or seven, and I have treated as many as ten teeth with abscesses, with the gums covered with spongy fistulous openings.

Under modern conditions of bottle-fed babies, impure foods, and crowded city life, a large number of children begin to need dental work in the second and third years, and often some of the teeth are lost by the fourth year.

One who is not familiar with such cases can imagine the great strain upon the system due to septicaemia that quickly follows the death of one or more pulps in a child's mouth. I leave it to you to picture the condition of such a child when it reaches the age to enter the kindergarten —if it lives that long—sickly, anaemic, irritable, stupid.

Every child compelled by law to attend school has the *right* to be protected from the spread of disease through the germ-laden breaths of the filthy mouths of such children.

In working for very young children I have found that if the teeth are polished once a month and given reasonable care at home no cavities will form.

In studying over the most economical way in which to care for the teeth of school children, I believe that the following will bring the best results, because the work is begun before or about the time of the eruption of the first permanent molar, which under present conditions is more often lost than any other tooth in the mouth, which loss is irreparable to the whole system of the child.

Have the teeth of all children filled, when necessary, before they enter the kindergarten.

Have a daily morning cleaning of the teeth as part of the exercises in the kindergarten.

Mark children upon their oral hygiene just as upon reading or spelling.

Arrange games that will develop strong, correctly-shaped jaws, such as lifting weights with the teeth or rapid running with tightly closed lips.

Give all the children in the kindergarten and first grade monthly prophylaxis. This last sounds formidable, but it could easily be managed at a small expense by having an auto-van fitted as a dental office to carry four dental chairs and an office desk. The force would consist of one dentist with four dental nurses and an assistant to sterilize the instruments. The van could be moved from school to school; the work in each school requiring part of a day only.

The electricity for the dental engines and sterilizer can be furnished by the engine of the van. Water could be connected at any hydrant and the waste pipe could be connected with the sewer.

The dentist in charge would oversee the work—mark the school cards, send any unusual cases to the stationary dental infirmary, and lecture at Mother meetings.

The result of such work would be magical. Epidemics of colds, measles, whooping cough, etc., would be almost unknown and the amount of school work each room could accomplish would be greatly increased. This would be one of the best ways to fight tuberculosis.

The final argument for such a method is that the school should teach the children the most valuable lessons in life—physical development and keeping the gateway to the body clean—by repeated lessons, just as it teaches them to read.

The Southern California Dental Association has been trying to educate the people since 1901. The question is, have we tried hard enough and in the right way? Are there more perfect first molars now or not? Also, what is being done to banish children's diseases? To be sure, when a child has measles or whooping cough he is sent home, but it is not often kept in quarantine, so he plays around. This would not be so serious a matter if only the child in school years were affected, but it is the child under school age, the baby brother of six months (who is on the bottle) or the little sister of two years, either of whom may not possess great resistance to disease. In such a case the fever may run such a course that malnutrition with lowered vitality will arrest the developing permanent teeth. Hypoplasia of the first permanent molar often results, decreasing its value fifty per cent.

With healthy children, a monthly polishing of the teeth and proper home care is sufficient, but since many children are below normal in health, it needs watchful care to save the first molar. In such cases, the fissures when the tooth first appears may be polished with an orange-wood stick and pumice, then dried with alcohol and cement flowed in, filling it nearly even with the cusps. When the occluding tooth comes into position, this will gradually wear down. Later, if the fissures darken and any softening is found, remove all caries and extend the cavity with a shallow undercut to the ends of the fissures and fill with copper amalgam.

I believe that there is no preventive measure of greater value than these shallow copper amalgam fillings in unhealthy mouths.

You all know my views with regard to silver amalgam fillings in the mouths of youth. There is only one place I use them and that is in the mesial cavity of the first molar before the bicuspid comes into position and I am not sure that they are satisfactory there.

The perfectors of the gold inlay have done great service in giving us an easy and as early perfect way of repairing the first molar, taking into consideration the fact that no repair can be as satisfactory as healthy tooth structure. However, in its use great care must be taken in preserving correct points of contact.

Where the pulp of the tooth is involved, one must be most conservative and exhaust every resource to keep it alive until root formation is complete. Here the X-Ray is our valuable aid in watching the progress of the treatment. Every ounce of skill we possess should be marshaled to our assistance in the care of such cases. Every rule and every preventive detail of sterilization should be followed in filling the root canals.

But above and beyond all, let us build up a plan to prevent this loss of the first molar in youth and save the future dentist the intricate and nerve-racking task of saving these forlorn hopes. Let us do our part in this, children's year, by teaching every mother in America to watch for the arrival of the first molar after a child is five years old and to have the mouth in proper array for this, the most valued tooth, our "First Line of Trench" in dentistry!

What is to hinder the dentists of America from adopting the most successful movement of the day? Why can not we, too, start a drive—a drive to save the first molar—the innumerable first molars. Why can not the Southern California Dental Association ask the numerous children's year committee to devote at least one week this fall to the education of the mothers of America, to emphasize the value of and teach them to save, the first permanent molar?—*Pacific Dental Gazette,* May, 1919.

